# Good long-term functional outcomes after rotationplasty despite osteoarthritis in the (pseudo)knee

**DOI:** 10.1016/j.ocarto.2025.100644

**Published:** 2025-06-26

**Authors:** G.G.J. Krebbekx, F.F. Smithuis, M.J.C. Duivenvoorden, R. Hemke, I.N. Sierevelt, G.R. Schaap, J.A.M. Bramer, G.M.M.J. Kerkhoffs, F.G.M. Verspoor

**Affiliations:** a Department of Orthopedic Surgery and Sports Medicine, Amsterdam UMC, Amsterdam Movement Sciences, University of Amsterdam, Meibergdreef 9, Amsterdam, the Netherlands; b Amsterdam UMC University of Amsterdam, Department of Radiology and Nuclear Medicine, Meibergdreef 9, Amsterdam, the Netherlands; c Xpert Clinics, Orthopedic Department, Laarderhoogtweg 12, Amsterdam, the Netherlands; d Spaarnegasthuis, Orthopedic Department, Spaarnepoort 1, Hoofddorp, the Netherlands; e Amsterdam Movement Sciences, Rehabilitation & Development, Amsterdam, the Netherlands; f Cancer Center Amsterdam, Amsterdam, the Netherlands

**Keywords:** Rotationplasty, Osteoarthritis, Sarcoma

## Abstract

**Objective:**

Rotationplasty is a surgical procedure primarily performed in patients with malignancies around the knee. Altered gait mechanics after surgery, such as reduced flexion of the (pseudo)knee and ipsilateral hip and changes in ground reaction forces, may predispose patients to osteoarthritis (OA) in the lower extremities. This study evaluated the long-term prevalence of OA and its association with pain and daily functioning.

**Method:**

Rotationplasty survivors who underwent surgery between 1980 and 2002 in Amsterdam received radiographic assessment of the (pseudo)knee, contralateral ankle, and both hips (weight-bearing mortise, lateral and AP views). OA was graded using the Kellgren-Lawrence scale. Functional outcomes, pain, quality of life, and sports participation were evaluated with the FAOS, AOFAS, and Harris Hip Score questionnaires. Statistical analyses included t-tests, Mann-Whitney U, chi-square, and Fisher's exact tests.

**Results:**

Thirty patients (mean age 49.4 ​± ​9.2 years; mean follow-up 32.4 ​± ​4.6 years) participated. Moderate-to-severe OA was found in 43 ​% of ipsilateral (pseudo)knees, 10 ​% of contralateral ankles, 33 ​% of ipsilateral hips, and 11 ​% of contralateral hips. Osteophytes were most common in the anterior tibial and subtalar regions (20 ​%), and joint space narrowing was most frequent in the subtalar (20 ​%) and medial tibiotalar (13 ​%) regions. Functional scores were generally favorable. The presence of osteoarthritis in the pseudo-knee was significantly associated with longer follow-up time.

**Conclusion:**

Functional outcomes after rotationplasty are well preserved over time, despite a higher prevalence of osteoarthritis in the (pseudo)knee compared to the contralateral ankle as a long-term consequence of the procedure.

## 1 Introduction

Since 1981, rotationplasty has been an alternative to transfemoral amputation for treating (malignant) tumors around the knee or addressing proximal femoral focal deficiencies [1]. This surgical procedure involves removing the affected femur segment, rotating the lower limb 180°, and reattaching it to the femur. The sciatic nerve is preserved, and the ankle joint functions as a (pseudo)knee, facilitating more natural gait mechanics compared to amputees. Advances in (neo)adjuvant therapies have improved sarcoma survival rates [2,3], making long-term outcomes such as the development of osteoarthritis (OA) and its impact on daily function and quality of life (QoL) increasingly relevant.

Patients with below-the-knee amputations have been reported to develop symptomatic OA in the ipsilateral knee and have a 50 ​% higher risk of ipsilateral hip OA compared to healthy individuals [4,5]. In patients with above-knee amputation, both hips and the contralateral knee showed signs of OA, with a threefold increased OA risk compared to those with below-knee amputation, 5–47 years after amputation [[5], [6], [7]]. Similarly, rotationplasty patients may have an increased risk of developing OA, representing a significant long-term complication as OA is associated with reduced QoL, functional limitations, and increased pain [8].

Gait deviations in rotationplasty patients, such as reduced flexion in the pseudo-knee and ipsilateral hip and altered ground reaction force patterns, contribute to joint asymmetry [[9], [10], [11], [12]]. This may increase OA risk in the ipsilateral and contralateral ankles and hips [10,[13], [14], [15]] as altered joint loading due to gait deviations is a risk factor for OA development [[16], [17], [18]].

Existing studies, albeit of limited sample size (7–22 participants), have reported signs of OA in the ipsilateral ankle and both hips, 5–25 years after rotationplasty [[19], [20], [21], [22], [23]]. Reports on the ipsilateral ankle vary, with OA observed in 5–100 ​% of ipsilateral tibiotalar or subtalar joints [19,20,22,23]. Only one study described the severity of OA in 17 patients and found mild OA in 13 ​% of ipsilateral hips and 3 ​% of contralateral hips with limited pain [21]. Severe degenerative changes have been noted in isolated cases, but functional loss due to OA remains underreported.

Given that OA is a progressive condition [16,24], concerns arise about whether rotationplasty patients may develop OA decades after the procedure, potentially accompanied by additional complaints such as pain and functional limitations. Rotationplasty is considered an alternative to above-knee amputation, particularly in the context of oncologic treatment, when autograft or allograft reconstructions are not feasible due to tumor involvement in vital structures [25,26]. As patients are increasingly involved in choosing between treatment options, both of which carry lifelong consequences, understanding long-term musculoskeletal outcomes is crucial to support informed decision-making. The impact of OA as a complication following rotationplasty remains insufficiently studied, making long-term follow-up studies essential to evaluate its potential significance.

This study aims to assess OA outcomes in the Amsterdam rotationplasty cohort, focusing on radiographic prevalence of OA in the ipsilateral (pseudo)knee compared to the contralateral ankle. Additionally, we assess OA in both hips. The study further explores the specific anatomical distribution of ankle OA and analyzes the relationship between ipsilateral OA and pain, as well as its impact on daily functioning and physical limitations. We hypothesize that rotationplasty patients, particularly older individuals, may show a higher prevalence of OA in the rotationplasty ankle and ipsilateral hip compared to the contralateral side, potentially resulting in functional impairment and increased pain.

## 2 Methods

This cross-sectional study was approved by the Medical Ethics Committee of the Amsterdam UMC on August 14, 2020 (reference number MEC.2020_018), adhering to the Declaration of Helsinki. This study is registered in the Dutch national trial register (NL72453.018.20). Conducted at a tertiary referral academic hospital specialized in oncological orthopedic diseases, the research was carried out in collaboration with two Amsterdam-based centers: Amsterdam UMC and Onze Lieve Vrouwe Gasthuis (OLVG).

### 2.1 Participants

Patient records from rotationplasty procedures performed for any indication between 1980 and 2002, ensuring a minimum follow-up period of 20 years, were reviewed for eligibility (n ​= ​70). There were no exclusion criteria.

### 2.2 Procedure

Following informed consent, eligible patients were invited to the Amsterdam UMC (location AMC) outpatient clinic for clinical examination, radiological diagnostics, and functional assessment by use of a patient-reported outcome measure.

### 2.3 Imaging

Radiographic OA assessments included mortise and lateral views of the rotationplasty (pseudo)knee and contralateral ankle, as well as anteroposterior and lateral views of both hips. For the pseudo-knee, weight-bearing radiographs were obtained with the prosthesis removed and the ipsilateral limb bearing weight on the table, while the contralateral limb remained on the ground. This setup allowed for a proper weight-bearing mortise and lateral view of the ipsilateral ankle. Radiographs of the contralateral ankle and both hips were taken with the exo-prosthesis in place, following standard weight-bearing positioning protocols.

All images were independently assessed for OA severity by a board-certified musculoskeletal radiologist and an orthopedic surgeon (GMMJK), both blinded to clinical data aside from the presence of a rotationplasty. The Kellgren-Lawrence (KL) grading scale was used to evaluate OA, ranging from 0 (no OA) to 4 (severe OA), based on osteophyte formation and joint space narrowing at defined anatomical locations [27]. OA was considered present at KL grade 2 or higher [27,28]. Any discrepancies in grading were resolved by a third independent reviewer (GGJK).

### 2.4 Clinical outcome measurements

Participants’ standing height and weight were measured while wearing their prosthesis, and the body mass index (BMI) was calculated (kg/m^2^).

Participants completed online questionnaires within a week before their outpatient clinic visit. No OA-specific functional questionnaires exist for rotationplasty, so the (pseudo)knee, morphologically an ankle, was assessed using the Foot and Ankle Outcome Score (FAOS) and the American Orthopedic Foot & Ankle Society (AOFAS) score [29]. FAOS is a patient-reported, ankle-specific functional outcome measure to evaluate symptoms, pain, Activities of Daily Living (ADL), sports/recreation participation, and QoL. The AOFAS is an ankle-specific physician-reported score assessing pain, function, and alignment. Hip impairments were evaluated using the Harris Hip Score [30], a validated multidimensional patient-reported outcome measure covering pain, walking, daily activities, and hip joint motion [31]. All scores range from 0 ​= ​maximum disability to 100 (no disability. Validated Dutch versions were employed, and reference data from age-matched healthy populations were used for comparison [[32], [33], [34], [35], [36]]. FAOS reference data were derived from the Danish population [37], AOFAS data from the German population [38], and Harris Hip Score from Australian and Canadian populations [39].

### 2.5 Statistical analysis

Data analysis was performed using SPSS Statistics for Windows, Version 28.0 (IBM Corp., Armonk, NY, 2021). Normality was assessed visually and confirmed to follow a normal distribution. Continuous data were expressed as means (SD) and categorical data as numbers with percentages. The McNemar test assessed the difference in prevalence of OA between rotationplasty side and the contralateral side. Two-sample t-tests were used to compare the FAOS and AOFAS outcomes to previously reported reference values, while a one-sample *t*-test was applied to compare the Harris Hip Score with reference values due to the absence of standard deviations. On the rotationplasty side, the association between OA and BMI, age, follow-up duration, and questionnaire outcomes were assessed using a Mann-Whitney *U* test. To assess associations with condition (malignant vs. benign), radiotherapy (with vs. without), and sex, we used the chi-square test or Fisher's exact test when expected cell counts were <5. A p-value <0.05 was considered statistically significant. No adjustment for multiple parallel testing was made due to the exploratory nature of the study.

Interrater reliability was evaluated using the dichotomic Cohen's kappa (with 95 ​% confidence interval). A kappa value of 0 indicates no agreement beyond chance, while a kappa value of 1 represents perfect agreement [40]. Additionally, the absolute agreement rate was calculated. A post-hoc power analysis was conducted for the primary outcome, prevalence of OA in the (pseudo)knee, using nQuery v8.5.1, as a sample size calculation could not be performed due to the lack of prior research on the topic.

## 3 Results

**Fig. 1:**
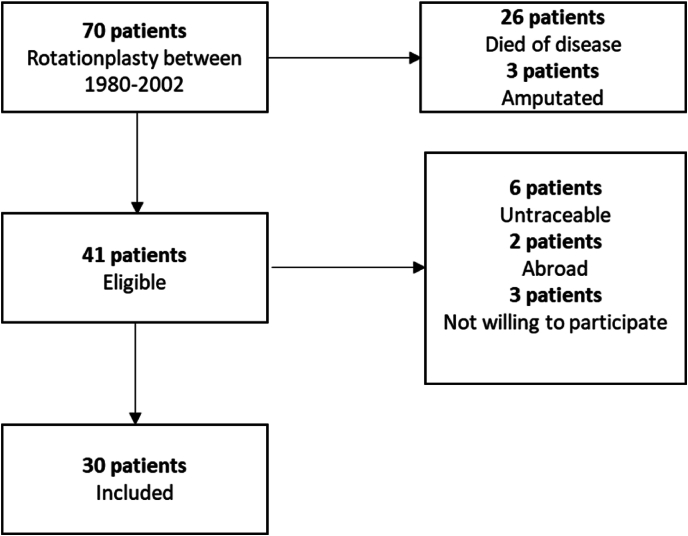
Inclusion.

Of the 70 patients who had a rotationplasty, 26 had passed away, three patients needed an amputation, and six were lost to follow-up. Of the remaining 35 patients, two lived abroad, two declined to participate due to radiation exposure, and one due to concerns about COVID-19, leaving 30 patients for inclusion (Fig. 1). One patient provided data only from the rotationplasty side due to prior clinical imaging (mortise and X-hip of the rotationplasty side) performed a few months earlier. This patient opted not to repeat X-rays for the contralateral side, and the previous imaging was used for comparison. Another patient, who had a total hip replacement for OA on the contralateral side, participated only in the ankle and rotationplasty hip assessments.

### 3.1 Patient characteristics

The mean follow-up of participants was 32.4 (SD 4.8) years after rotationplasty, with a mean age of 49.4 years (SD 9.2) at the time of assessment. All patients had a Winkelmann type A1 rotationplasty [41]. The indications for rotationplasty were as follows: Twenty-three patients (77 ​%) were diagnosed with osteosarcoma, two patients (7 ​%) with Ewing sarcoma, two patients (7 %) with malignant fibrous histiocytoma, one patient (3 ​%) with malignant giant cell tumor of the bone, one patient (3 ​%) with severe hemangioma, and one patient (3 ​%) with proximal femoral focal deficiencies. The mean BMI was 25.5 (SD 3.8). Eighteen patients (60 ​%) had the procedure on the right side, and 12 (40 ​%) on the left side. Two patients (7 ​%) required walking aids (Table 1).

**Table 1:** Characteristics of rotationplasty patients.

Variable	Mean (SD)
Age in years during test day	49.4 (9.2)
Age in years during procedure	17.0 (9.8)
Follow-up time in years	32.4 (4.8)
Body weight (kg)	80.9 (13.9)
Height (cm)	177.9 (10.2)
BMI (kg/m^2^)	25.5 (3.8)
	**Number of patients (percentage)**
Rotationplasty side	R 18 (60) L 12 (40)
Gait aid	2 (6.7)
Hip replacement	Contralateral side: 1 (3.3)

SD: standard deviation, BMI: body mass index, R: right, L: Left.

### 3.2 Prevalence of osteoarthritis

**Fig. 2:**
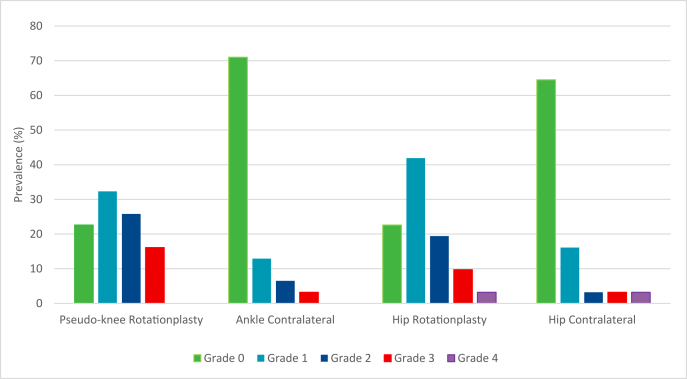
Prevalence of OA per KL-grade.

**Fig. 3:**
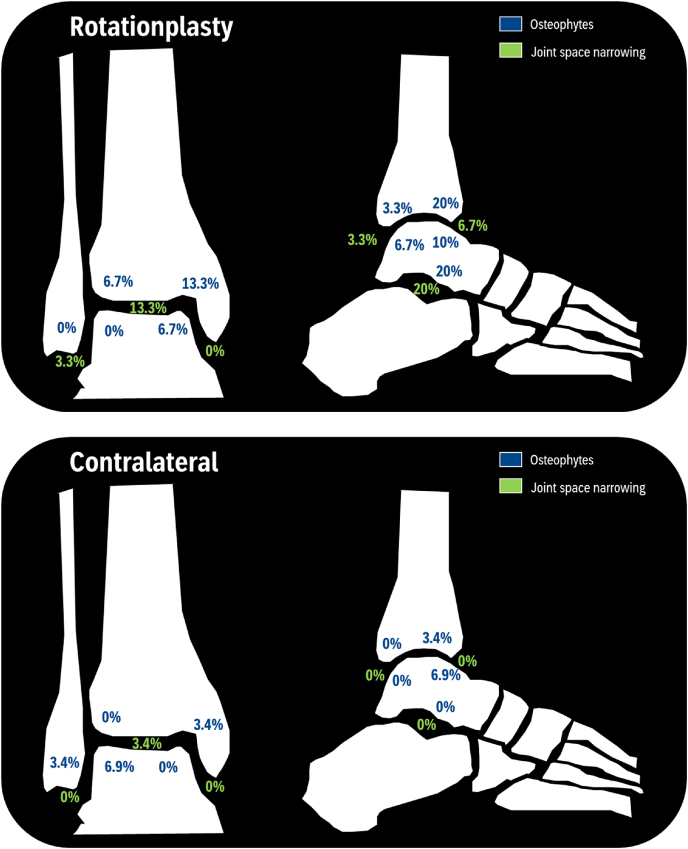
Prevalence of ankle OA.

Overall, 13 of patients (43 %) showed moderate to severe findings of OA on the ipsilateral (pseudo)knee (KL-2 n ​= ​8, KL-3 n ​= ​5, KL-4 n ​= ​0), compared to 3 (10 %) of the contralateral ankle (KL-2 n ​= ​3, KL-3 n ​= ​1, KL-4 n ​= ​0) (*p* ​= ​*0.02*). Osteophytes were most common in the anterior tibial (20 %), subtalar (20 %), and medial tibial (13 %) regions on the rotationplasty site, with joint space narrowing mainly observed in the subtalar (20 %) and medial tibiotalar (13 %) regions. On the contralateral side, osteophytes were most frequently observed in the lateral talar (7 %) and anterior talar (7 %) regions (Table 2, Fig. 2, Fig. 3).

**Table 2:** Osteoarthritis in rotationplasty patients.

	Rotationplasty N (%)	Contralateral N (%)	*P*-value
**Ankle**			
KL-grade			0.02
≥2	13 (43.3)	3 (10.3)	
<2	17 (56.7)	26 (89.7)	
KL-grade			
0	7 (22.6)	22 (71.0)	
1	10 (32.3)	4 (12.9)	
2	8 (25.8)	2 (6.5)	
3	5 (16.1)	1 (3.2)	
4	0 (0)	0 (0)	
**Hip**			
KL-grade			0.11
≥2	10 (33.3)	3 (10.7)	
<2	20 (66.7)	25 (89.3)	
KL-grade			
0	7 (22.6)	20 (64.5)	
1	13 (41.9)	5 (16.1)	
2	6 (19.4)	1 (3.2)	
3	3 (9.7)	1 (3.2)	
4	1 (3.2)	1 (3.2)	

KL: Kellgren-Lawrence, N: number of participants.

**Fig. 4:**
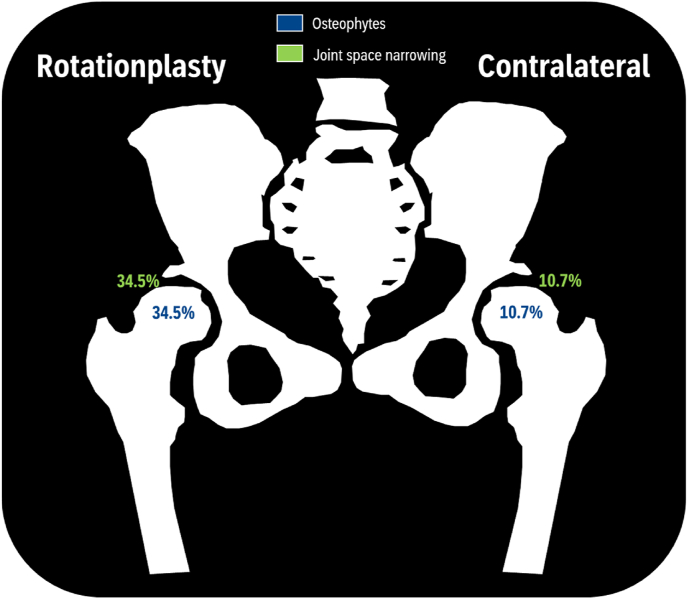
Prevalence of hip OA.

Overall, 10 patients (33 ​%) showed moderate to severe findings of OA in the ipsilateral hip (i.e., the hip on the rotationplasty leg) with KL-2 in 6 cases, KL-3 in 3, and KL-4 in 1. In comparison, 3 patients (11 ​%) had OA in the contralateral hip (KL-2 n ​= ​1, KL-3 n ​= ​1, KL-4 n ​= ​1). This difference was not statistically significant (*p* ​= ​*0.11*) (Table 2, Fig. 2, Fig. 4).

Post-hoc power analysis revealed 83.5 ​% power to detect the described prevalence difference in ankle OA, with a current sample size of 30 patients.

The interobserver reliability for the total OA KL-scores showed a kappa of 0.81 (95 ​% CI: 0.68–0.93), with an absolute agreement of 87 ​%.

### 3.3 Questionnaires

For the (pseudo)knee, no significant differences were observed in the FAOS; symptoms, ADL, and QoL compared to reference data. The total AOFAS scores did not differ significantly for the (pseudo)knee, and the contralateral side compared to reference data. Pain scores for the (pseudo)knee were significantly higher (less pain) compared to reference data (FAOS: mean difference ​= ​4.8, *p* ​= ​*0.05*, AOFAS: mean difference ​= ​3.4, *p* < *0.001*). Rotationplasty patients had reduced sports participation (FAOS: mean difference ​= ​−43.4, *p* < *0.001*) as well as lower (pseudo)knee function (AOFAS: mean difference ​= ​−5.5, *p* < *0.001*), compared to the reference data (Table 3).

**Table 3:** Questionnaires rotationplasty patients.

	Mean (SD)	Reference data mean (SD)	Mean difference (CI95 ​%)	*P*-value
**FAOS:**				
N	30	345		
Symptoms	87.5 (10.2)	85.3 (17.4)	2.2 (−2.0; 6.3)	0.30
Pain	92.6 (11.8)	87.8 (18.8)	4.8 (0.0; 9.6)	0.05
ADL	87.9 (13.3)	91.1 (17.4)	−3.20 (−8.4; 2.1)	0.23
Sports	40.5 (26.0)	83.9 (25.4)	−43,4 (−53.4; −33.3)	<0.001
QoL	72.5 (20.0)	81.2 (24.8)	−8.7 (−16.6; −0.8)	0.06
**AOFAS:**				
N	30	112		
R total	89.7 (8.3)	91.1 (2.3)	−1.4 (−4.5; 1.7)	0.37
C total	91.8 (7.8)	91.1 (2.3)	0.7 (−2.2; 3.6)	0.63
R Pain (40 points)	38.0 (4.1)	34.6 (1.5)	3.4 (1.8; –4.9)	<0.001
R Function (50 points)	41.7 (6.9)	47.2 (1.0)	−5.5 (−8.1; −2.9)	<0.001
R Alignment (10 points)	10 (0)	9.3 (0.4)	N/A	N/A
**Harris hip:**				
N	30	NA		
R total	96.4 (8.2)	97.6	−1.2 (4.3; –1.9)	0.45
C total	98.2 (4.4)	97.6	0.6 (−1.1; 2.2)	0.49

N: number of participants, R: rotationplasty side, C: contralateral side, ADL: activity of daily living, QoL: quality of life, N/A: not applicable, NA: not available.

The mean Harris Hip Scores for both hips did not differ significantly from reference data (Table 3).

### 3.4 Association

For the rotationplasty (pseudo)knee, only a significant positive association was found between the presence of OA and a longer follow-up time (*p* ​= ​*0.05*) (no adjustment for multiple parallel testing). No significant association was found between the presence of OA in the (pseudo)knee and diagnosis (benign or malignant), treatment with or without radiotherapy, sex, BMI, age on test day, FAOS (pain, symptoms, ADL, sports and QoL), and AOFAS (total, pain and function). In the hips, no significant associations were found between the presence of OA and diagnose (benign or malignant), treatment with or without radiotherapy, sex, BMI, age at test day, FU-time, Harris Hip score, and pain (Table 4).

**Table 4:** Association patient characteristics and outcome measures.

Rotationplasty Ankle	OA	No OA	*P*-value
Total number (N (%))	13	17	
Diagnose N (%)			0.49
*Malignant*	13 (100.0)	15 (88.2)	
*Benign*	0 (0.0)	2 (11.8)	
Radiotherapy N (%)			1.00
*With*	1 (7.7)	1 (5.9)	
*Without*	12 (92.3)	16 (94.1)	
Sex N (%)			0.71
*Female*	7 (54.0)	8 (47.1)	
*Male*	6 (46.0)	9 (52.9)	
BMI	24.5 (2.6)	26.3 (4.4)	0.41
Age-test day	52.0 (8.1)	47.4 (9.7)	0.09
FU-time	34.4 (4.0)	30.8 (4.9)	0.05∗
FAOS Pain	87.2 (15.6)	96.7 (5.2)	0.06
FAOS symptoms	83.8 (11.8)	90.3 (8.0)	0.13
FAOS ADL	82.1 (17.5)	92.4 (6.6)	0.16
FAOS sports	35.8 (27.0)	44.1 (25.4)	0.41
FAOS QoL	63.5 (24.7)	79.4 (12.3)	0.06
AOFAS total	88.2 (10.5)	90.8 (6.3)	0.60
AOFAS pain	37.7 (4.4)	38.2 (3.9)	0.72
AOFAS function	40.6 (8.6)	42.5 (5.5)	0.60

**Rotationplasty hip**	**OA**	**No OA**	***P*-value**

Total N (%)	10	20	
Diagnose N (%)			1.00
*Malignant*	9 (90.0)	19 (95.0)	
*Benign*	1 (10.0)	1 (5.0)	
Radiotherapy N (%)			0.54
*With*	0 (0.0)	2 (10.0)	
*Without*	10 (100.0)	18 (90.0)	
Sex N (%)			1.00
*Female*	5 (50.0)	10 (50.0)	
*Male*	5 (50.0)	10 (50.0)	
BMI	25.6 (1.9)	25.5 (4.5)	0.48
Age-test day	51.7 (9.8)	48.3 (8.9)	0.45
FU-time	32.1 (5.0)	32.5 (4.8)	0.83
Harris hip	99.3 (1.6)	95.0 (9.8)	0.16
Pain	0 (0)	0.2 (0.4)	0.20

Described in mean (SD) unless otherwise stated, ∗Significant, N: number of participants, BMI: body mass index, FU: follow-up, ADL: activity of daily living, QoL: quality of life.

## 4 Discussion

This study evaluates the long-term outcomes of OA in patients after rotationplasty surgery, with a mean follow-up of 32.4 years. Moderate to severe OA was observed in 43 ​% of (pseudo)knees and 33 ​% of ipsilateral hips, with the (pseudo)knee showing prominent OA in the talocrural and subtalar joints. Despite these radiographic OA findings, functional outcomes, as measured by the FAOS (symptoms, pain, ADL, and QoL), total AOFAS, and Harris Hip Scores, remained unaffected. However, the FAOS indicated reduced sports participation, while the AOFAS revealed decreased function of the (pseudo)knee compared to reference data. Nonetheless, the presence of OA in the (pseudo)knee was only positively associated with follow-up duration, and functional outcomes did not significantly differ between patients with and without (pseudo)knee OA.

This study has several limitations. The small sample size, due to the rarity of rotationplasty procedures, limits both statistical power and generalizability. However, this cohort represents the largest group evaluated for OA outcomes after rotationplasty and includes the longest reported follow-up, with minimal selection bias as nearly all eligible patients participated. The use of basic radiographic imaging (AP, mortise, and lateral views) restricted the detail of OA assessment. Although advanced imaging could have provided more comprehensive insights, we prioritized minimizing radiation exposure. Despite this, OA was detectable, as demonstrated by high interrater reliability. Another limitation is the absence of OA-specific functional questionnaires tailored to rotationplasty patients. Instead, ankle-specific tools were used, despite the unique biomechanics of the (pseudo)knee. Additionally, questionnaire responses may have been influenced by factors unrelated to OA, such as the original disease or surgical intervention, introducing potential confounding. The absence of a control group and reliance on reference data further limit direct comparison to the general population. Future research should aim to include larger cohorts, employ advanced imaging modalities, and incorporate matched control groups to strengthen generalizability.

The (pseudo)knee had a considerably higher OA prevalence than the 9.2 ​% reported for the talocrural joint in healthy populations. In contrast, the contralateral ankle showed an OA prevalence of 10.4 ​%, aligning more closely with the healthy population [42]. Our findings surpass those of earlier studies, which reported slight signs of OA in 29 ​% in the (pseudo)knee and 0 ​% in the contralateral ankle, 4–15 years after rotationplasty [19,20,22,23]. Subtalar joint OA was present in 20 ​% of (pseudo)knees, significantly higher than in the contralateral side, and much greater than the 0.4 ​% reported in healthy populations [42]. Loss of mobility in the subtalar joint among rotationplasty patients was previously noted by Gottsauner-Wolf et al. [22]. The biomechanics of ankle OA development remain poorly understood [43,44]. As OA progresses, talus orientation shifts, and the subtalar joint compensates to maintain hindfoot alignment in early to intermediate stages [45]. This adaptation may contribute to subtalar OA progression, although the unique use of the (pseudo)knee makes it more complex. Notably, the equine's position maintained during walking shifts the load to the posterior talar region, reducing the loaded surface area and increasing mean and peak pressures [9], despite the reduced push-off forces observed in the (pseudo)knee during gait [46]. The equine's position resembles the *relevé* posture in ballet dancers, where the subtalar joint is forced into a locked position. Such forced positioning, beyond the joint's normal range of motion, can result in microtrauma. This phenomenon has been associated with higher OA scores in the subtalar joints of ballet dancers compared to non-dancers [47].

Possibly due to a longer follow-up time, this study found a higher prevalence of OA in the ipsilateral hip compared to the 13 ​% reported 25 years after rotationplasty [21]. The OA prevalence was also higher compared to the estimated prevalence of hip OA in the general European population (12.59 ​%), which could be due to differences in age between our cohort and the reference population [48]. Another possible explanation involves gait-related factors. Specifically, the cumulative hip moment has been associated with OA development in the frontal plane [18]. However, studies have shown that the frontal hip moment on the rotationplasty side is significantly reduced compared to the unaffected side [11]. Nevertheless, the cumulative hip moment could still be elevated due to repetitive movements over time. Another gait-related risk factor for hip OA is a larger hip flexion angle during early stance [49]. While hip flexion during early stance has not been previously described in rotationplasty patients, a greater range of motion during walking was observed, with increased flexion during swing and greater extension in stance [11,50], possibly contributing to the higher hip OA prevalence.

OA is generally linked to decreased QoL due to functional limitations and restrictions [8,51]. However, a significant number of patients with radiographic evidence of OA have no clinical symptoms [52]. Which is in line with our cohort; despite radiographic OA, patients showed no significant functional deficits on the Harris Hip Score. These results align with Ackman et al., who found no significant differences in function or pain between rotationplasty patients and healthy controls 25 years post-surgery [15]. Functional results for the (pseudo)knee, measured by the AOFAS and FAOS, were also good in terms of pain, symptoms, alignment, and QoL, but patients reported slightly reduced function and limited sport participation compared to reference data. This aligns with findings that radiographic OA is not linked to pain severity or disability in patients with chronic ankle complaints but is associated with functional loss in talocrural OA [53]. However, in rotationplasty patients, OA was not associated with function loss and sports participation, suggesting that this may be more attributable to the procedure itself rather than the effect of OA.

This study found no significant association between sex, BMI, age, and OA, contrasting with their recognized roles as risk factors for OA [24,[54], [55], [56], [57], [58]]. For example, female sex is a known risk factor for OA, particularly after the age of 40, an age group representative of our study population [57,58]. Nonetheless, in this study, no association between sex and OA was observed. Instead, follow-up time after rotationplasty was associated with OA, suggesting that the altered anatomy and gait biomechanics, including the unique use of the ankle as a pseudo-knee, may play a more dominant role in OA development, while factors such as age and BMI appear to be less influential in this population. Future research should assess the impact of gait biomechanics on OA progression in rotationplasty patients, as this remains unexplored.

## 5 Conclusion

In conclusion, over three decades after rotationplasty, patients showed a significantly higher prevalence of OA in the (pseudo)knee (particularly in the talocrural and subtalar joints) compared to the contralateral ankle. Rotationplasty patients demonstrated satisfactory outcomes compared to reference data, with comparable scores for symptoms, ADL, QoL, and total AOFAS, good pain scores. Only scores related to sports participation and functional performance were reduced. Notably, the presence of OA in the (pseudo)knee was associated with longer follow-up duration but did not correspond to worse functional outcomes compared to those with OA. Future studies should investigate how long-term biomechanical factors, such as altered gait patterns, contribute to OA development in rotationplasty patients.

## Ethical review committee statement

The study has been carried out following medical ethical committee of the Amsterdam University Medical Centre, Amsterdam, The Netherlands.

## Author contributions

FV, GGJK, GMMJK, GS, and JB initiated the study. FV, GGJK, and MD worked on the conceptual design of the study. GS and GGJK identified and invited eligible participants. FV, FS, GGJK, GMMJK, GS, JB, and RH collected the existing data. GGJK, IS, and MD have worked on the database. GGJK and IS have worked on statistical analyses. GGJK, IS, and MD wrote the manuscript under supervision of FV, FS, GMMJK, GS, JB, and RH. All authors critically reviewed and revised the manuscript and contributed to interpretation of the data. All authors read and approved the final version of the manuscript. GGJK acts as guarantor and accepts full responsibility for the finished work and/or the conduct of the study, had access to the data, and controlled the decision to publish.

## Conflict of interest

Each author certifies that there are no funding or commercial associations (consultancies, stock ownership, equity interest, patent/licensing arrangements, etc.) that might pose a conflict of interest in connection with the submitted article related to the author or any immediate family members.
